# Damiana New Aphrodisiac

**Published:** 1875-08

**Authors:** J. J. Caldwell

**Affiliations:** Baltimore, Md.


DAMIANA.—*By J. J. Caldwell, M.D., Baltimore, Md.*—I now desire to invite your attention to a plant—new to the medical world—called in the Indian tongue "Damiana" (a powerful aphrodisiac), and found on the western borders of Mexico.

In giving the history of this wonderful and beautiful little plant, I regret to say that I cannot present, as yet, its technical name or true classification; but I am very well satisfied, from quite an extended experience with the tincture and extract of this plant, of its powerful influence over the urino-genital organs of both sexes, as in moderate doses it increases the flow of urine, as well as the sexual appetite, as my cases reported in this article will show.

CASE I.—Mr. H., of Carroll county, Md., aged 70, called, June, 1874, to be treated for impotency. As he had just married his fourth wife, he manifested great anxiety concerning his weakened powers. I advised him to try the benefit of Faradic current through the genito-sacral plexus. His occupation being such as to require almost constant travel, he was unable to follow my orders in this particular. I then placed him upon the strong tincture of damiana, in tablespoonful doses three or four times a day, which resulted in a marked improvement in his procreative powers, so that, after a few weeks' continued use of the remedy, he reported himself "well able to enjoy sexual congress, of course observing a moderation due in a man of his age."

CASE II.—In October, 1874, Mr. M., of Baltimore, informed me that his wife, after a severe illness, with mental trouble, lost all her sexual appetite, her age being 40. Her health being well re-established, I resorted to this nervine tonic, with very happy results. Her husband, being robust and vigorous, as a matter of experiment, used the same remedy in tablespoonful doses twice a day, resulting in excessive and almost ungovernable sexual desire; and this has proven true in several other cases of vigorous constitution upon which I have experimented.

CASE III.—Col. L., of Baltimore, aged 55, called, December, 1874, suffering from general debility of the genito-urinary organs, attributed to the excessive use of alcohol. He, too, was placed upon the tincture of damiana, and followed it up faithfully for a month or six weeks, with the very best results, greatly increasing the secretion of his urine, besides improving his sexual ability.

CASE IV.—Mr. K. has been under my care for over a year, suffering from stricture of the urethra, with extreme irritation of the bladder (sympathetic.) The stricture was treated by galvanic electrolysis successfully, by placing an elastic insulated steel-pointed catheter, attached to the negative pole of the galvanic battery, applying the same with gentle pressure, while the positive pole, a zinc plate four inches square, covered with a napkin saturated with salt water, was placed over the sacral spine—these applications made on alternate days, with fifteen minutes application each. After the stricture had been absorbed and removed by this mode of treatment, the irritability of the parts yielded to the use of the tincture of damiana, in moderate doses, twice a day.

The foregoing is a report of a few typical cases that have come under my observation, and are given as an introduction of this new remedy, and for what they are worth, solely with the view of calling the attention of the profession to the virtues of this pretty little plant, culled from the prolific soil of the wilds of Old Mexico—a field no doubt teeming with a wealth of unknown medicines, waiting for the progress of our noble and searching science to penetrate and grasp her hidden treasures.

I would recommend the tincture and fluid ext. of damiana. I prefer the fluid extract, as it is less bulky, more positive in its effects, and more reliable and uniform, as proven in cases now under my care. Indeed, this remedy seems to have a specific effect upon all of the organs of the pelvis, giving increased tone and activity to all of the secretions in that vicinity.—*Virginia Medical Monthly.*

